# Neuro-inflammation induced by lipopolysaccharide causes cognitive impairment through enhancement of beta-amyloid generation

**DOI:** 10.1186/1742-2094-5-37

**Published:** 2008-08-29

**Authors:** Jae Woong Lee, Yong Kyung Lee, Dong Yeon Yuk, Dong Young Choi, Sang Bae Ban, Ki Wan Oh, Jin Tae Hong

**Affiliations:** 1 College of Pharmacy and CBITRC, Chungbuk National University 12, Gaesin-dong, Heungduk-gu, Cheongju, Chungbuk 361-763, Korea; 2 Department of Anatomy and Neurobiology, College of Medicine, University of Kentucky Lexington, KY 40506, USA

## Abstract

**Background:**

Alzheimer's disease (AD) is characterized by extensive loss of neurons in the brain of AD patients. Intracellular accumulation of beta-amyloid peptide (Aβ) has also shown to occur in AD. Neuro-inflammation has been known to play a role in the pathogenesis of AD.

**Methods:**

In this study, we investigated neuro-inflammation and amyloidogenesis and memory impairment following the systemic inflammation generated by lipopolysaccharide (LPS) using immunohistochemistry, ELISA, behavioral tests and Western blotting.

**Results:**

Intraperitoneal injection of LPS, (250 μg/kg) induced memory impairment determined by passive avoidance and water maze tests in mice. Repeated injection of LPS (250 μg/kg, 3 or 7 times) resulted in an accumulation of Aβ_1–42 _in the hippocampus and cerebralcortex of mice brains through increased β- and γ-secretase activities accompanied with the increased expression of amyloid precursor protein (APP), 99-residue carboxy-terminal fragment of APP (C99) and generation of Aβ_1–42 _as well as activation of astrocytes *in vivo*. 3 weeks of pretreatment of sulindac sulfide (3.75 and 7.5 mg/kg, orally), an anti-inflammatory agent, suppressed the LPS-induced amyloidogenesis, memory dysfunction as well as neuronal cell death *in vivo*. Sulindac sulfide (12.5–50 μM) also suppressed LPS (1 μg/ml)-induced amyloidogenesis in cultured neurons and astrocytes *in vitro*.

**Conclusion:**

This study suggests that neuro-inflammatory reaction could contribute to AD pathology, and anti-inflammatory agent could be useful for the prevention of AD.

## Background

Alzheimer's disease (AD) is a progressive neuro-psychiatric disorder. The major neuropathological hallmarks of AD are the formation of senile plaques (SPs) following neurofibrillary tangles (NFTs) which cause neuronal degeneration and synaptic loss. SPs are extracellular deposits of fibrillar and amorphous aggregates of amyloid beta-peptide (Aβ) whereas NFTs are intracellular fibrillar aggregates of the microtubule-associated protein tau that exhibit hyperphosphorylation. The formation of SPs and NFTs in brain regions such as the entorhinal cortex, hippocampus, basal forebrain and amygdala impaired learning and memory functions [1]. AD brains also exhibit a number of pathological abnormalities, including a profound loss of synapses, reactive gliosis, and inflammatory processes [2].

The brain has an endogenous immune system that is coordinated by immunocompetent cells such as microglia. The brain is also vulnerable to constitutive defense responses, such as inflammation [3,4]. The inflammation associated with the brain, neuro-inflammation, differs from that found in the periphery. Although edema and neutrophil invasion, typical features of inflammation, is not seen in the AD brain, tissue levels of inflammatory mediators including cytokines, chemokines, oxygen free radicals and reactive nitrogen species, are altered [5,6].

Numerous reports have indicated that neuro-inflammatory process contributes to the pathogenesis of AD. Study performed in transgenic animals suggest that neuro-inflammation plays an important role in the process of cerebral amyloid deposition [7]. It has been shown that inflammatory cytokines such as Interleukin (IL)-1β, IL-6, Tumor necrosis factor-αgTNF-α) or Transforming growth factor-β (TGF-β) can augment APP expression [8,9] and Aβ formation [10]. It was also reported that cytokines are able to transcriptionally upregulate β-secretase mRNA, protein and enzymatic activity [11]. β-secretase is a key rate-limiting enzyme that initiates Aβ formation [12]. Without β-secretase, Aβ synthesis is either abolished or considerably reduced [13]. Moreover, McGeer and Rogers proposed possible therapeutic effects of anti-inflammatory agents on the patients with AD [14]. Inflammatory mediators present in AD lesions are thought to stimulate underlying key events of the pathological cascade that result in increased Aβ production with recruitment and activation of microglial cells [15].

Many persons with AD die with systemic inflammation such as a lung or bladder infection. The systemic inflammation will lead to the generation of circulating cytokines, which will have in turn an impact on the central nervous system [16]. Furthermore, it was also reported that intraperitoneal injection of lipopolysaccharide (LPS) induces cognitive impairment in mice [17,18]. However, underlying mechanisms involved in LPS induced cognitive impairment are not known. To investigate the impact of systemic inflammation on memory impairment and its role in cortical amyloid formation and deposition, mice were intraperitoneally injected with LPS to generate systemic inflammation, and then investigated for the possible mechanisms of LPS-induced memory impairment and amyloidogenesis *in vivo *and *in vitro*.

## Methods

### Animals

Male ICR mice (Damool Science, Korea) weighing 25–30 g and Sprague-Dawley rats weighing 200–300 g, were used in all experiments. Animals were maintained in accordance with the National Institute of Toxicological Research, Korea Food and Drug Administration guidelines for the care and use of laboratory animals. Animals were housed in two cages (five per cage) and in a 22 ± 2°C and 45~65% relative humidity environment under a 12-hr light/12-hr dark cycle (8:00 a.m. ~8:00 p.m.). All animals had free access to food (Samyang Foods, Seoul, Korea) and water. The anti-inflammatory sulindac sulfide (3.75 or 7.5 mg/kg) was given orally for 3 weeks prior to the injection of LPS in *in vivo *study.

The mice were randomly divided within each cage and injected intraperitoneally with either 250 μg/kg of Lipopolysaccharide (LPS) or sterile saline (0.9% NaCl). For all experiments, LPS (*Escherichia coli*, serotype 055:B5, Sigma, St. Louis, MO, USA) was used to induce an inflammatory response and was injected once on day 1 of behavioral testing. All injections were administered 4 hrs prior to testing. This allows enough time for the development of neuro-inflammation expressing central IL-1β gene (most notably in circumventricular organs, meningeal tissue, and choroid plexus) at this dose and similar doses of intraperitoneal LPS [19].

### Behavioral test

#### 1. Passive avoidance test (Step-through test)

The passive avoidance test is a widely accepted simple and rapid means of memory testing. Passive avoidance response was determined using a "step-through" apparatus (Med Associated Inc., St. Albans, VT, USA), which consisted of an illuminated and dark compartment (each 20.3 × 15.9 × 21.3 cm) adjoining each other through a guillotine door. Floors were constructed of 3.175 mm stainless steel rods set 8 mm apart. The test was conducted for 2 consecutive days at the same time each day. On the first day (learning trial) each mouse was placed in the illuminated compartment facing away from the dark compartment. Once the mouse enters completely into the dark compartment, it receives an electric shock (1 mA, 3 s) through the stainless steel grid floor. The amount of time it took for the mouse to enter into the dark compartment was recorded automatically, and described as step-through latency. On the second day (testing trial), the same test procedure was followed. When the mouse did not enter the dark compartment within 300s, the test was terminated and a latency of 300s was recorded.

#### 2. Water maze test

The water maze test was performed as described by Morris et al. [20] using the SMART-CS (Panlab, Barcelona, Spain) program and equipment. A circular pool (height: 35 cm, diameter: 100 cm) was filled with water, dyed black by dissolving food colorings and maintained at 22~25°C. An escape platform (height: 14.5 cm, diameter: 4.5 cm) was then submerged 0.5~1 cm below the surface of the water in the northeastern quadrant of the pool. On training trials, the mice were placed in the pool of water and allowed to remain on the platform for 10 s and were then returned to their cage during the second-trial interval. The mice that did not find the platform within 120 s were placed on the platform for 10 s at the end of trial. 24 hrs after 6 trials (two times per day for 3 days), mice were given LPS. Four hrs after the treatment of LPS (designated as day 1), they were allowed to swim until they sought the escape platform. Escape latency, escape distance, swimming speed and swimming pattern of each mouse was monitored for 3 days (1 time/day) by a camera above the center of the pool connected to a SMART-LD program (Panlab, Barcelona, Spain).

### Tissue preparation

After the behavioral tests, animals were perfused with PBS under inhaled diethyl ether anesthesia. Brains were immediately collected, stored at -20°C, and separated into cortical and hippocampal regions. The brain regions (hippocampus and cerebralcortex) were immediately stored at -80°C before an assay of secretase activities, Aβ_1–42 _level as well as western blotting.

### Astrocyte culture

As described elsewhere [21,22], 2-day-old rat pups were ice-anesthetized and decapitated. After the skin was opened and the skull was cut, the brain was released from the skull cavity. After washing with PBS, the cerebrum was separated from the cerebellum and brain stem, and the cerebral hemispheres were separated from each other by gently teasing along the midline fissure with the sharp edge of forceps. The meninges were gently peeled from the individual cortical lobes and the cortices were dissociated by mechanical digestion [using the cell strainer (BD Biosciences, Franklin Lakes, NJ, USA)] with Dulbecco's modified Eagle's medium (DMEM) containing F12 nutrient mixture (Invitrogen, Carlsbad, CA). The resulting cells were centrifuged (1,500 rpm, 5 mins), resuspended in serum-supplemented culture media, and plated into 100 mm dishes. Serum-supplemented culture media was composed of DMEM supplemented with F12, FBS (5%), NaHCO_3 _(40 mM), penicillin (100 units/ml), and steptomycin (100 μg/ml). The cells were incubated in the culture medium in a humidified incubator at 37°C and 5% CO_2_for 9 days. At confluence (9 days), the flask was subjected to shaking for 16–18 hrs at 37°C. The cultures were treated for 48 hrs with cytosine arabinoside and the medium was replaced with DMEM/F12HAM containing 10% FBS. The monolayer was treated with 1.25% trypsin-EDTA for a short duration after which the cells were dissociated and plated into uncoated glass coverslips. The astrocyte cultures formed a layer of process-bearing, GFAP-positive cells. The purity of astrocyte cultures was assessed by GFAP-immunostaining. Under these conditions, we can assume that over 95% of the cells were astrocytes. The cultured cells were treated with LPS or TNF-α or IFN-γ for 24 hrs, and cells were harvested for the assay of Aβ and western blotting.

### Embryonic neuronal cell culture

The Sprague-Dawley pregnant rats were sacrificed by cervical dislocation and the embryos were removed on the 18^th ^day of gestation. The embryonic brain tissues were mechanically dissociated into individual cells in NEUROBASAL medium (Invitrogen, Carlsbad, CA, USA). The resulting cells were centrifuged (1,500 rpm, 5 min), resuspended in NEUROBASAL medium containing B-27 supplement (Invitrogen, Carlsbad, CA), L-glutamine (0.5 mM), penicillin (100 units/ml), steptomycin (100 μg/ml) and plated into 60 mm dishes. The culture media was changed every 2 days. Greater than 90% of the cells in these cultures were neurons as assessed by cell morphology and immunostaining with mouse monoclonal antibodies against neurofilaments (1: 5,000). 7 day cultured cells were treated with LPS or TNF-α or IFN-γgor 24 hrs, the cells were harvested for the assay of Aβ and western blotting.

### Western blotting

Brain tissues and cells were homogenized with protein extraction solution (PRO-PREP™, Intron Biotechnology, Korea), and lysed by 60 min incubation on ice. The lysate was centrifuged at 15,000 rpm for 15 min. Equal amount of proteins (40 μg) were separated on a SDS/10% or 15%-polyacrylamide gel, and then transferred to a polyvinylidene difluoride (PVDF) membrane (GE Water & Process technologies, Trevose, PA, USA). Blots were blocked for 2 hrs at room temperature with 5% (w/v) non-fat dried milk in Tris-Buffered Saline Tween-20 [TBST: 10 mM Tris (pH 8.0) and 150 mM NaCl solution containing 0.05% tween-20]. After a short wash in TBST, the membrane was incubated at room temperature with specific antibodies. Rabbit polyclonal antibodies against iNOS and COX-2 (1: 1,000 dilution, Cayman Chemical, Ann Arbor, MI, USA), APP (1:500 dilution, ABR, Golden, CO, USA), BACE1 (1:500 dilution, Sigma, St. Louis, MO, USA), C99 (1:500 dilution, Sigma, St. Louis, MO, USA) and mouse monoclonal antibody against phospho-ERK (1:500 dilution, Santa Cruz Biothechnology Inc. Santa Cruz, CA, USA) were used in the study. The blot was then incubated with the corresponding conjugated anti-rabbit or mouse immunoglobulin G-horseradish peroxidase (1:2,000 dilutions, Santa Cruz Biotechnology Inc. Santa Cruz, CA, USA). Immunoreactive proteins were detected with the BM Chemiluminescence blotting substrate (Roche applied science, Mannheim, Germany).

### Immunohistochemistry and immunofluorescence

Mice were euthanized with diethyl ether and perfused with 0.1 M PBS then with 4% paraformaldehyde. The brains were collected from mice following perfusion and immediately fixed in 4% paraformaldehyde for 24 hrs. The brains were transferred successively to 10%, 20% and 30% sucrose solutions. Subsequently, brains were frozen on a cold stage and sectioned in a cryostate (40 μm-thick). Sections were treated with endogenous peroxidase (3% H_2_O_2 _in PBS), and then with 0.01 M PBS blocking buffer containing 10% bovine serum albumin in PBS for 40 min. Then the sections were incubated with rabbit polyclonal antibody against Aβ_1–42 _(1:2,000 dilution, Covance, Berkeley, CA, USA), and iNOS and COX-2 (1: 1,000 dilution, Cayman Chemical, Ann Arbor, MI, USA), overnight. After the incubation, sections were washed in PBS and incubated with the biotinylated secondary antibodies (ABC kit, Vector Laboaratories, Burlingame, CA) for 30 min. The sections were washed with PBS, incubated with the avidin-biotin complex (Vector Laboratories, Burlingame, CA) for 30 min, and visualized by chromogen DAB (Vector Laboratories, Burlingame, CA) reaction. The sections were dehydrated in ethanol, cleared in xylene, and mounted with permaunt (Fisher Scientific, Hampton, NH). For the detection of cellular location of Aβ_1–42_, we did an immunofluorescence immunostaining. Sections were rinsed in 0.01 M PBS buffer. After washing in PBS, the sections were incubated for 1 hr at room temperature with 10% bovine serum albumin diluted in PBS. The sections were incubated overnight at 4°C with Rabbit Polyclonal Aβ_1–42 _antibody (1:2000 dilution, Covance, Berkeley, CA, USA). After washing in PBS, the sections were washed and incubated with Alexa Fluro 568 conjugated Rabbit Polyclonal antibody (1:200 dilution, Molecular Probe, Carlsbad, CA, USA) for 2 hrs at room temperature. Next, the sections were incubated with DAPI for 15 min at 37°C. Finally, the sections were rinsed, mounted on slides, and coverslipped for fluorescence microscopy and photography using ApoTome microscope (Carl Zeiss, Inc., Thornwood, NY, USA). For detection of apoptotic cell death in tumor tissue, the paraffin embedded sections were then incubated in the mixture of labeling solution (450 μl) and enzyme solution (50 μl) for 1 hr at 37°C and washed 3 times in 0.1 M PBS for 5 min each according to manufacturer's instructions. Next, the sections were incubated with DAPI for 15 min at 37°C. Finally, the sections were rinsed, mounted on slides, and coverslipped for fluorescence microscopy (DAS microscope). Positive TUNEL stains were recorded by counting the number of positively stained DAPI in the definite area.

### α-, β- and γ-secretase activity assays

The total activities of α-, β- and γ-secretase present in cortical and hippocampal regions were determined using a commercially available α-secretase activity kit (R&D systems, Wiesbaden, Germany), β-secretase fluorescence resonance energy transfer (BACE 1 FRET) assay kit (PANVERA, Madison, USA) and γ-secretase activity kit, (R&D systems, Wiesbaden, Germany) according to the manufacturer's instructions, respectively. Each tissue was homogenized in cold 1 × cell extraction buffer (a component of the kit) to a final protein concentration of 1 mg/ml.

To determine α (or γ)-secretase activity, 50 μl of lysate was mixed with 50 μl of reaction buffer. The mixture was incubated for 1 hr in the dark at 37°C after 5 μl of substrate was added. Substrate conjugated to the reporter molecules EDANS and DABCYL was cleaved by α (or γ)-secretase and released a fluorescent signal. This fluorescence was measured using a Fluostar galaxy fluorometer (excitation at 355 nm and emission at 510 nm) equipped with Felix software (BMG Labtechnologies, Offenburg, Germany). The level of α (or γ)-secretase enzymatic activity was proportional to fluorescence with the intensity of fluorescene which was expressed as fluorescence units.

To determine β-secretase, 10 μl of lysate was mixed with 10 μl of BACE1 substrate (Rh-EVNLDAEFK-Quencher). The reaction mixture was then incubated for 1 hr at room temperature in a black 96-microwell plate. The reaction was stopped by adding 10 μl of BACE1 stop buffer (2.5 M sodium acetate). Fluorescence was determined using a Fluostar galaxy fluorometer (excitation at 545 nm and emission at 590 nm) equipped with Felix software (BMG Labtechnologies, Offenburg, Germany). Enzyme activity was linearly related to fluorescence increases, and the activity was expressed as fluorescence units. All controls, blanks and samples were run in triplicate.

### Measurement of Aβ level

Lysates of brain tissue prepared as described in the Western blotting section were obtained through protein extraction buffer containing protease inhibitor. Media from neuronal cell culture was collected, then briefly spun to remove cell debris and mixed with 4-(2-aminoethyl)-benzene sulfonyl fluoride serine protease inhibitor. Aβ_1–42 _and Aβ_1–40 _levels were determined using specific ELISAs (IBL, Immuno-Biological Co., Ltd., Japan). In short, 100 μl of sample was added into the precoated plate and was incubated overnight at 4°C. After washing each well of the precoated plate with washing buffer, 100 μl of labeled antibody solution was added and the mixture was incubated for 1 hr at 4°C in the dark. After washing, chromogen was added and the mixture was incubated for 30 mins at room temperature in the dark. After the addition of stop solution, the resulting color was assayed at 450 nm using a microplate absorbance reader (Sunrise™, TECAN, Switzerland).

### Statistics

The experimental results were expressed as mean ± S.E. A one-way analysis of variance (ANOVA) was used for multiple comparisons followed by Dunnett. Differences with P < 0.05 were considered statistically significant.

## Results

### LPS induced memory impairment

**Figure 1:**
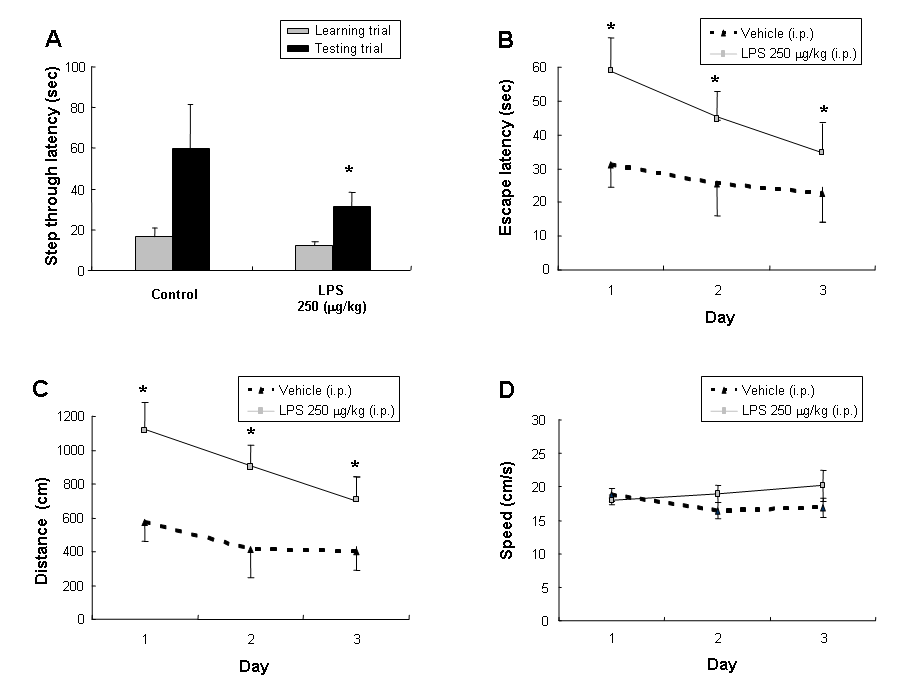
Effect of LPS on step-through type passive avoidance test (**A**) and water maze test (**B, C, D**). **(A)**, Each value is mean ± S.E. from 7–9 mice. *Significantly different from control (p < 0.05). Memory function was determined by the escape latencies (**B**, sec), distance (**C**, cm) and speed (**D**, cm/sec) for 3 days at 4 hr (designated 1 day) after administration of LPS. Each value is mean ± S.E. from 7–9 mice. *Significantly different from control (p < 0.05).

In the passive avoidance test, at the learning trial (day 0), mice of all groups entered the dark compartment, and there were no significant differences among the animals. However, in the testing trial (day 1), the mice which received a single intraperitoneal injection of LPS (250 μg/kg) showed a significantly reduced step-through latency compared to those injected with vehicle (Fig. 1A). In the water maze test, the mice exhibited progressively decreased escape latency by the training (3 days after training; 2 times/day, total of 6 times training), and the escape latency at the end of training (7^th ^escape latency) to the platform was about 403 ± 44 cm and 19 ± 1 s (data not shown). LPS was then administered into the mice. Similar to the result in the step through test, LPS-treated mice showed longer escape latency (the time required to find the platform) and escape distance (the distance swam to find the platform) than the control group (Fig. 1B). This tendency continued throughout the 3-day trial period although the difference between the two groups was getting smaller. The LPS-treated group traveled a further distance to reach the platform than the control group did (Fig. 1C). It is considered that these differences between the two groups reflected the difference in memory function since there was not much difference in swimming speed (Fig. 1D).

### LPS induced Aβ generation in mice brains

**Figure 2:**
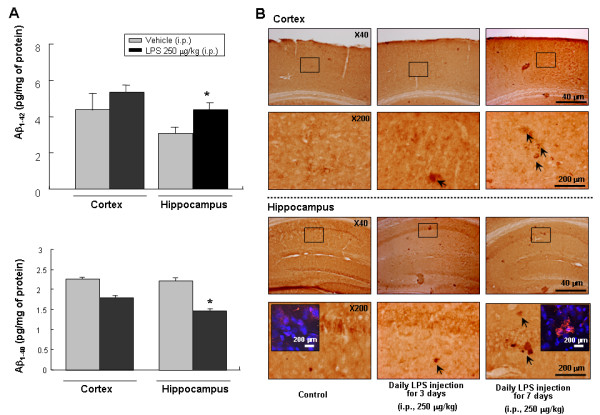
**Effect of LPS on Aβ accumulation in the cortex and hippocampus.** The levels of Aβ_1–42 _and Aβ_1–40 _(**A**) were assessed by using a specific Aβ ELISA as described in the Materials and methods section. Values measured from each group of mice were calibrated by amount of protein and expressed as mean ± S.E. (n = 5) *Significant different from control (p < 0.05). Immunostaining of Aβ_1–42 _in the cortex and hippocampus (**B**). Mice were injected intraperitoneally with either 250 μg/kg LPS or sterile saline (0.9% NaCl) daily for 3 or 7 days before sacrifice. Forty μm-thick sections of brains from mice were incubated with rabbit polyclonal anti-Aβ_1–42 _antibody and counterstained with hematoxylin. Arrow indicates Aβ_1–42 _accumulation which is clearly higher in the cerebral cortex and hippocampus of LPS-treated mouse and was the highest in the mouse treated with daily injection for 7 days. Figure in box shows the intracellular accumulation of Aβ_1–42 _(detected anti-Aβ_1–42 _immunofluroscene staining after DAPI staining the cells) in the pyramidal neurons of the hippocampus at the high magnification. Arrow bar indicates accumulation of Aβ_1–42_.

A single intraperitoneal injection of LPS increased the Aβ_1–42 _level in the cortex and hippocampus (Fig. 2A). In contrast, a single intraperitoneal injection of LPS decreased the Aβ_1–40 _level in the cortex and hippocampus (Fig. 2A). To assess the pattern of Aβ_1–42 _deposition, we analyzed the Aβ_1–42 _immunoreactivity in the cortex and hippocampus following daily LPS injections for 3–7 days. An increase of Aβ_1–42 _immunoreactivity was observed in the LPS injected group compared to that of the control group (Fig. 2B). Aβ_1–42 _immunoreactivity progressively increased with the duration of LPS adminstration and was much more intense in the hippocampus compared to the control.

### LPS decreased α-secretase activity but increased βg and γ-secretase activities as well as expression of APP, BACE and C99 proteins in mice brains

**Figure 3:**
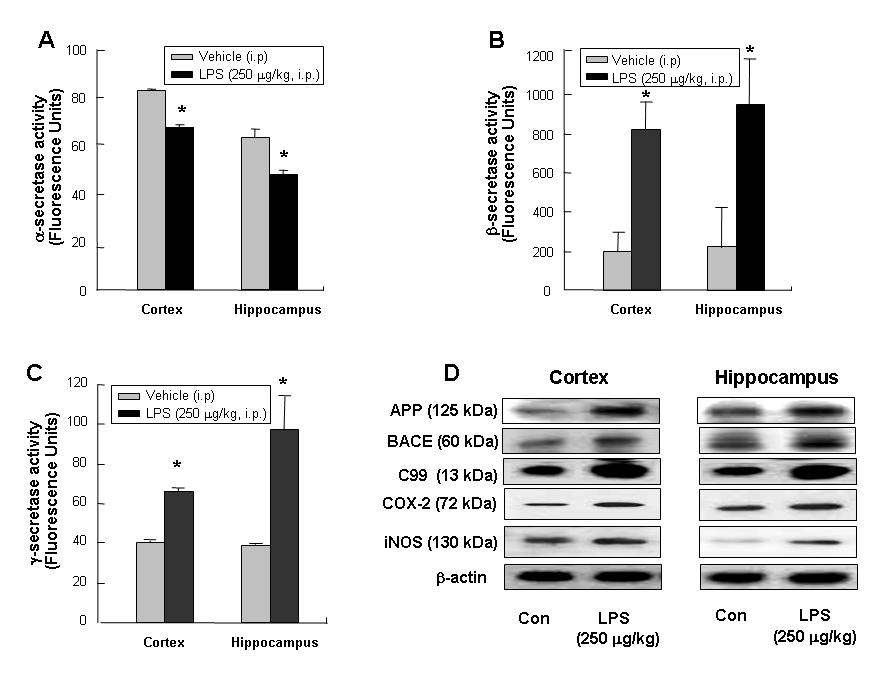
**Effect of LPS on secretase activities and amyloidogenic proteins expression.** The activities of α-, β-secretase (**A, B**) and γ-secretase (**C**) were assessed by using commercially available assay kits. Data represent mean ± S.E. (n = 5). *Significant different from control group (p < 0.05). The expression of APP, BACE and C99 (**D**) were detected by Western blotting using specific antibodies. Each blot is representative for five experiments. β-actin protein was used here as an internal control.

Following a single injection of LPS, the activities of β and γ-secretase in the cortex and hippocampus increased (Fig. 3B and 3C), whereas, the activity of α-secretase decreased in mice brains (Fig. 3A). Moreover, LPS treatment increased expression of APP, BACE and C99 accompanied with the increase of inflammatory proteins iNOS and COX-2 expression (Fig. 3D).

### Inflammatory agents promoted amyloidogenesis *in vitro*

**Figure 4:**
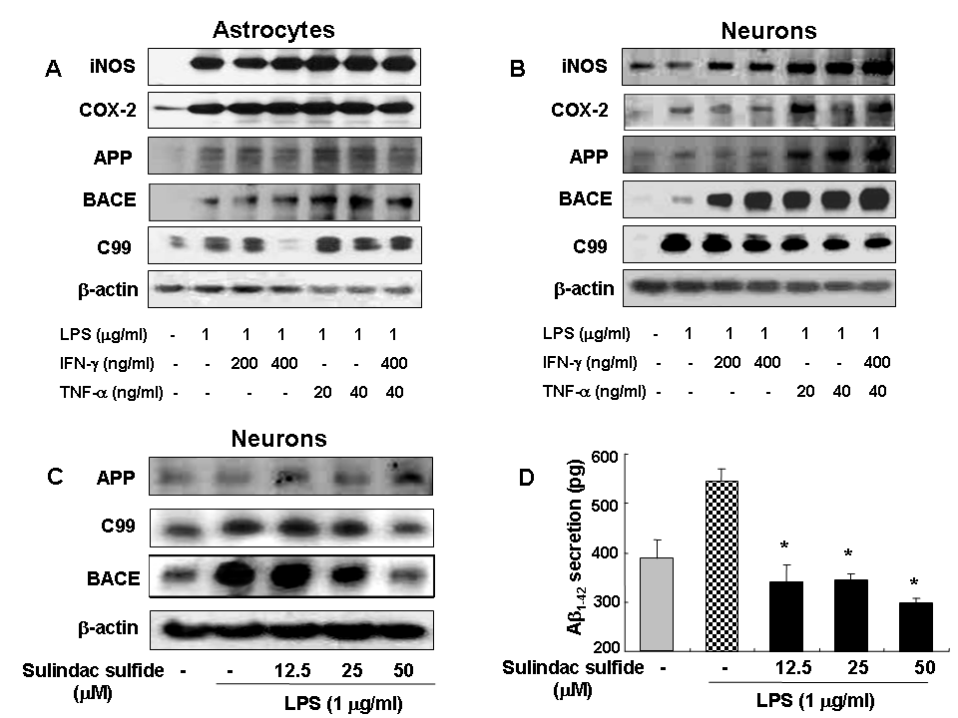
Effect of anti-inflammatory agents on expression of amyloidogenic proteins (**A**, **B**). The expression of APP, BACE and C99 were detected by Western blotting using specific antibodies in astrocytes (**A**) and neuronal cells (**B**). β-Actin protein was used as an internal control. Each blot is representative for five experiments. Sulindac sulfide inhibits expression of amyloidogenic proteins **(C) **and Aβ_1–42 _secretion (**D**) induced by LPS in cultured neuronal cells. Combined Sulindac sulfide (12.5, 25, 50 μM) and LPS treatment for 24 hr were used. **(C)**, The expression of APP, BACE and C99 in neuronal cells was detected by Western blotting using specific antibodies. β-Actin protein was used as an internal control. (**D**), Media were collected to determine an Aβ_1–42 _secretion by ELISA. Data represent mean ± S.E. of three experiments with duplicated. *Significant different from LPS treated group (p < 0.05).

It is known that microglia and astrocytes are major sources of neuro-inflammation. Moreover, recent data showed that neuronal cells also have cytokine receptors such as LPS receptor (toll like receptor) as well as TNF receptor [23,24]. Neurons may be directly involved in neuro-inflammation or indirectly via the interaction with microglia and astrocytes. In order to analyze the effect of LPS induced inflammation on amyloidogenesis in vitro, cultured astrocytes from rat pups and neuronal cells from rat embryos were used. Astrocytes lend both mechanical and metabolic support for neurons, regulating the environment in which they function. Interferron-gamma (IFN-γ) and tumor necrosis factor-alpha (TNF-α) as well as LPS were treated to induce an inflammatory reaction. Similiar to the *in vivo *results, inflammatory stimuli concomitantly increased expression of amyloidogenic proteins (such as APP, BACE and C99) accompanied with the increase of expression of inflammatory proteins (such as COX-2 and iNOS) in both astrocytes (Fig. 4A) and neuronal cells (Fig. 4B). These results further indicate amyloidogenic pathway could be promoted by neuro-inflammatory stimulation in *in vitro *and *in vivo*.

### Anti-inflammatory drug inhibited LPS-induced amyloidogenesis and memory impairment

**Figure 5:**
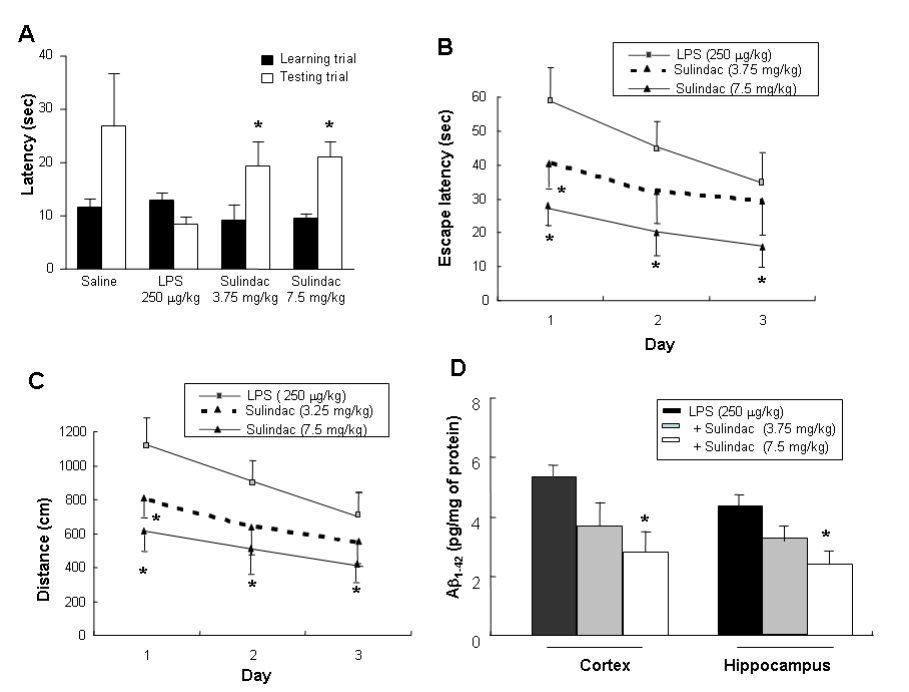
Effect of sulindac sulfide on the LPS-induced memory impairment (**A-C**) and elevated Aβ_1–42 _level (**D**). Sulindac sulfide was pretreated for 3 weeks by oral administration. For the passive avoidance performance test, mice were trained one time. At 24 hr later, mice were given LPS (250 μg/kg, i.p.). After 4 hr treatment of LPS, the latency period was measured. Each value is means ± S.E. from 15 mice. *Significantly different from LPS treated control (p < 0.05). **(B-C)**, Mice were pretreated with Sulindac sulfide for 3 weeks, and then trained for 3 days (2 times/day, 6 times training), and then LPS (250 μg/kg, i.p.) was administered into mice. Memory function was determined by the escape latencies (cm, **B**) and distance (sec, **C**) at 4 hr (designated day 1). Each value is means ± S.E. from 15 mice. **D**, The levels of Aβ_1–42 _were assessed after finishing the behavioral tests by using a specific Aβ_1–42 _ELISA. Values measured from each group of mice were calibrated by amount of protein and expressed as mean ± S.E. (n = 15) *Significant different from LPS treated group (p < 0.05).

The effect of sulindac sulfide, a COX-1, 2 non-selective drugs, *in vivo *and *in vitro *system was assessed. Sulindac sulfide has been known to decrease the Aβ secretion in N2a neuroblastoma cells stably transfected with human APP695 bearing the Swedish mutation [25]. As shown in Fig. 4C, the cells expressed low levels of APP, β-site APP cleavage enzyme (BACE) and C99 protein in an unstimulated condition, whereas, expression of BACE, APP and C99 proteins increased in response to LPS (1 μg/ml) after 24 hrs. Treatment with sulindac sulfide (12.5, 25, 50 μM) caused concentration-dependent decreases in LPS-induced BACE, and C99 expression in neuronal cells, but did not change the expression of APP. In addition, sulindac sulfide decreased LPS-induced Aβ_1–42 _secretion into culture media (Fig. 4D). Furthermore, oral pretreatment with sulindac sulfide (3.75 and 7.5 mg/kg) for 3 weeks suppressed memory impairment caused by LPS, and reduced increased Aβ_1–42 _levels (Fig. 5D) in concentration-dependent manners. This was evaluated with the passive avoidance test (Fig. 5A) and the water maze test (Fig. 5B and 5C). It is considered that sulindac sulfide may have an endogenous Aβ-lowering effect in neuronal cells, and suggests that inflammatory reaction could influence the amyloidogenesis, and thus could improve memory function.

### LPS caused neuronal cell death in the brain

**Figure 6:**
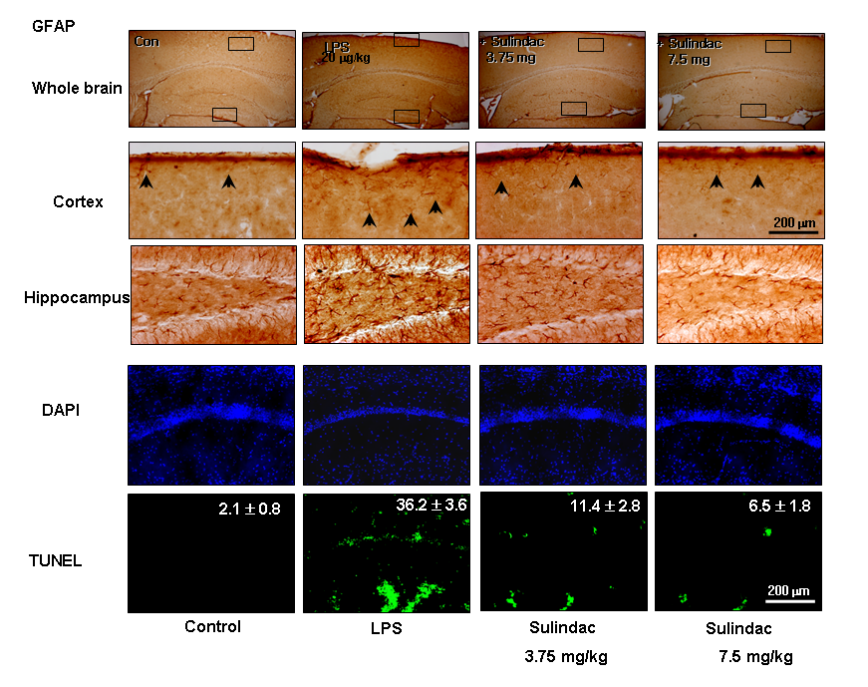
Effect of LPS on activation of astrocytes **(A) **and apoptotic cell death **(B)**. Mice were injected intraperitoneally with either 250 μg/kg LPS or sterile saline (0.9% NaCl) daily for 7 days before sacrifice. Forty μm-thick sections of brains from mice were immunostained with rabbit polyclonal anti-GFAP antibody for evaluation of activation of astrocytes. The broad distribution and deep intensity of GFAP reactive cells increased in the LPS injected mice brain. Each panel is representative of 6 animals. Apoptotic cell death was determined by DAPI staining and TUNEL assay. Apoptosis (%) was defined as the percentage of the number of TUNEL-positive cells per surface of unit. Values are mean ± S.E. (n = 6). *P < 0.05 indicates significantly different from LPS-treated cells.

To verify the relationship between LPS-induced accumulation of Aβ and neuronal cell death, we investigated the induction of cell death by LPS *in vivo*. Substantial increase of apoptotic cells was found in the hippocampus of LPS treated mice. A significant increase in the percentage of the number of apoptotic cells was detected in the LPS treated animals (36.2 ± 3.6%) verses the control (2.1 ± 0.8%). The percentage of the number of apoptotic cells in the brains of LPS treated animals was significantly reduced by the sulindac sulfide pretreatment. The values were 11.4 ± 2.8% (3.75 mg/kg), and 6.1 ± 1.8% (7.5 mg/kg), respectively (Fig. 6A).

The activation of astrocytes was analyzed by their immunoreactivity for GFAP which was more intensive in LPS treated mice brains than in controls. It was also reduced by sulindac sulfide pre-treatment (Fig. 6B).

## Discussion

Epidemiological and genetic evidences have shown that an inflammatory process may contribute to AD pathology. However, the exact relationship and mechanisms are not clear. Therefore, we tried to establish a convincing theoretical link between neuro-inflammatory reaction and amyloidogenesis. Our results demonstrated that systemic injections of LPS induced memory impairment. LPS also induced Aβ_1–42 _generation in both the cortex and hippocampus. In *in invo and in vitro *studies, expression of the genes involved in inflammation and in amyloidogenesis was also concomitantly increased by the LPS treatment. Moreover, the anti-inflammatory drug sulindac sulfide inhibited the LPS-induced memory impairment and amyloidogenesis. These results indicate that systemic inflammation induced by LPS could cause memory impairment through enhancement of amyloidogenesis.

Recent studies have demonstrated that LPS influences Aβ deposition [25], and anti-inflammatory agents prevent Aβ deposition [26]. Ibuprofen, a commonly used nonsteroid anti-inflammatory drug, decreased cytokine-stimulated Aβ production in human neuronal cells and astrocytes [27]. It also reduced Aβ levels and brain inflammation in Tg2576 AD mice [28]. Indometacin given to Tg2576 mice also reduced insoluble Aβ_1–42 _in the hippocampus [29]. Our previous data also showed a co-elevated expression of Aβ_1–42 _and COX-2 as well as IL-1 in presenlinin 2 mutant AD transgenic mice [30]. Our present study demonstrated that co-expression of inflammatory proteins COX-2 and iNOS, and amyloidogenic proteins BACE and C99 was higher in the LPS-treated mice brains, and LPS alone or LPS with IFN-γ or TNF-α treated cultured astrocytes and neuronal cells. However, the anti-inflammatory drug sulindac sulfide decreased the LPS-induced expressions of BACE and C99 as well as COX-2 and iNOS. These data indicate that expression of inflammatory proteins could be linked with expression of the proteins related with amyloidogenesis. We also found that LPS treated brains showed higher levels of Aβ_1–42 _but lower levels of Aβ_1–40_. It may be interesting to note that several other investigators demonstrated that Aβ_1–40 _could be cytoprotective. Kuperstein et al. reported that Aβ_1–40 _protects fetal rat brain from intrauterine ischemic stress [31]. Zou et al. also demonstrated that Aβ_1–40 _protects neurons from damage induced by Aβ_1–42 _in culture and in rat brains [32] via serving as an antioxidant molecule against metal-induced oxidative damage [33]. This increase of Aβ_1–42 _with concomitant decrease of Aβ_1–40_could be related with the elevation of the expression of amyloidogenic proteins in LPS-treated mice brains, and could also be involved in neuronal damages causing memory dysfunction. Very similar to our findings, Hauss-Wegrzyniak and Wenk showed that LPS induced extracellular deposition of beta-amyloid fibrils into the hippocampus [34]. Therefore, our results suggest that there is a close connection between amyloidogenesis and neuro-inflammation induced by systemic injection of LPS, and thus neuro-inflammation enhances Aβ generation which impairs memory function.

The way LPS induces amyloidogenesis is not clear. However, it could be related with the change in secretase activities. APP is first cleaved by β-secretase at its β-cleavage site generating a membrane bound C99 whose subsequent proteolysis by a second enzyme, γ-secretase produces Aβ_1–42_. Thus, we determined secretase activities. Consistent with the increasing effect on Aβ_1–42 _generation and expression of APP, BACE and C99, LPS treatment increased β- and γ-secretase activities. It has been shown that inflammatory cytokines IL-1β, IL-6, TNF-α and TGF-β augmented APP expression [8,9], and Aβ formation [10], and these processes may be related with the activation of transcriptional upregulation of β-secretase mRNA, protein and enzymatic activity [11]. It has also been observed that TNF-α, IL-1β and IFN-γ stimulate γ-secretase so as to control Aβ generation [35]. Sheng et al. also reported that the systemic injection of LPS increases APP expression and processing with accumulation of Aβ in APPswe transgenic mice [36]. LPS-induced increase of APP level in the present study is in agreement with the observation by Rogers et al. [37] who demonstrated that the primary inflammatory cytokine enhanced APP gene expression at the translational level through the well characterized IL-1 responsive element of APP mRNA. Taken together, these data indicated that LPS-enhanced inflammatory reactions could influence APP processing through the enhancement of β and γ-secretase activities, thereby affecting amyloidogenesis. Bandyopadhyay et al. showed that cytokine interleukin-1α stimulates non-amyloidogenic pathway by the alpha-secretase (ADAM-10 and ADAM-17) cleavage of APP in human astrocytes [38]. It was observed that IL-1β induced sAPPα release via α-secretase cleavage in neuroglioma U251 cells [39]. We also found that LPS decreased α-secretase. These findings suggest that one of other mechanism increasing of β-amyloid by LPS may be in part due to the inhibition of α-secretase activity.

The signals in LPS treatment induced amyloidogenesis could be involved with the activation of AP-1 since APP gene promoter contains potential activator protein-1 (AP-1) recognition site [40-42], and LPS could activate AP-1 activity [43]. Activation of the MAP kinase pathway may relay the amyliodogenesis signal as demonstrated in other studies [44,30,46] in which MAP kinase plays a role in neuro-inflammatory and neurodegenerative pathology of relevance to AD. In the present study, it was also found that phosphorylation of ERK, a type of MAP kinase, was elevated in the LPS treated group, and sulindac sulfide decreased the activation of ERK and Aβ_1–42 _secretion in neuronal cells (data not shown). These data indicate that ERK/AP-1 signal pathway may be important in the LPS-induced amyloidogenesis.

The consequence of elevated Aβ_1–42 _by LPS could cause neuronal cell death, and this may be associated with memory impairment. In fact, we found that LPS-treated mice brains showed increased number of cell death *in vivo*. However, treatment of sulindac sulfide inhibited LPS-induced neuronal cell death, suggesting that induction of neuronal apoptotic cell death by LPS may directly result from the induction of amyloidogenesis by neuro-inflammation. Noble et al. reported that acute systemic inflammation induces central mitochondrial damage and amnesic deficit in adult Swiss mice [47]. Sparkman [17], and Milatovic et al. [48] and Szczepanik and Ringheim [49] reported that intraperitoneal injections of LPS cause AD-like neuronal degeneration. We have also found that mutant presenilin 2 (a genetic AD model) mice brains showed increased inflammation and accumulation of Aβg accompanied by an increase of apoptotic neuronal cell death. LPS induced neuro-inflammatory signal activation (Cox-2 and ERK activation) could be involved in the LPS-induced neuronal cell death. Jang and Surh showed that beta-amyloid-induced apoptosis is associated with cyclooxygenase-2 up-regulation through activation of NF-κB, which is mediated by upstream kinases including ERK and p38 MAPK [50]. We also previously demonstrated that Bcl-2 overexpression protects neuronal cells against Aβ-induced cell death in differentiated PC12, and its protective effect was related to the reduction of Aβ-induced activation of p38 MAP kinase [51]. Even though the exact signal pathways in the LPS-induced neuronal cell death and amyloidogenesis are not clear, the increase of apoptotic neuronal cell death via the elevation of Aβ_1–42 _could be an important mechanism in LPS-induced memory impairment. The activation of astrocytes by treatment with LPS may induce several cytotoxic cytokines which could also hurt neighboring neuronal cells via directly killing mechanisms [52-54] or via elevation of Aβ_1–42 _[9,10]. In conclusion, systemic inflammation by treatment with LPS causes elevation of amyloidogenesis and neuronal cell death which finally result in memory impairment.

## Conclusion

In conclusion, our current study showed that systemic inflammatory stimuli elevated amyloidogenesis through activation of β- and γ-secretases accompanied with inhibition of α-secretase leading to elevated Aβ_1–42 _levels *in vivo *and *in vitro*. This co-elevated inflammation and amyloidogenesis resulted in neuronal cell death, and thus memory impairment. Moreover, the anti-inflammatory drug sulindac sulfide inhibited both amylodogenesis and neuro-inflammation which led to recovery effects on the LPS-induced memory impairment. Therefore, the present data suggest that systemic inflammation could be involved in the development and/or progression of AD, and anti-inflammatory drugs might be useful for the prevention of AD.

## Competing interests

The authors declare that they have no competing interests.

## Authors' contributions

JWL performed behavioral tests and some of ELISA assay, and performed western blotting, performed some of the statistical analyses, and prepared and wrote the manuscript. YKL performed the immunohistochemical staining, assessment of neuronal complement immunoreactivity, and helped to write the manuscript. DYC performed the immunohistochemical evaluation and reviewed the manuscript. DYY assisted with the data collection, was involved in the experimental design, and wrote and reviewed the manuscript. SBB and KWO assisted with the manuscript preparation, and discussed the behavioral and biochemical changes. JTH designed the studies, reviewed the data, and wrote the manuscript. All authors read and approved the final manuscript.

## References

[B1] Mattson MP, Maudsley S, Martin B (2004). A neural signaling triumvirate that influences ageing and age-related disease: insulin/IGF-1, BDNF and serotonin. Ageing Res Rev.

[B2] Pratico D, Trojanowski JQ (2000). Inflammatory hypotheses: novel mechanisms of Alzheimer's neurodegeneration and new therapeutic targets?. Neurobiol Aging.

[B3] Masliah E, LiCastro F (2000). Neurodegenerative dementias: clinical features and pathological mechanisms.

[B4] Neuroinflammation Working Group (2000). Inflammation and Alzheimer's disease. Neurobiol Aging.

[B5] Eikelenboom P, Bate C, Van Gool WA, Hoozemans JJ, Rozemuller JM, Veerhuis R, Williams A (2002). Neuroinflammation in Alzheimer's disease and prion disease. Glia.

[B6] McGeer EG, McGeer PL (2003). Inflammatory processes in Alzheimer's disease. Prog Neuropsychopharmacol Biol Psychiatr.

[B7] Guo JT, Yu J, Grass D, de Beer FC, Kindy MS (2002). Inflammation-dependent cerebral deposition of serum amyloid a protein in a mouse model of amyloidosis. J Neurosci.

[B8] Hirose Y, Imai Y, Nakajima K, Takemoto N, Toya S, Kohsaka S (1994). Glial conditioned medium alters the expression of amyloid precursor protein in SH-SY5Y neuroblastoma cells. Biochem Biophys Res Commun.

[B9] Buxbaum JD, Oishi M, Chen HI, Pinkas-Kramarski R, Jaffe EA, Gandy SE, Greengard P (1992). Cholinergic agonists and interleukin 1 regulate processing and secretion of the Alzheimer beta/A4 amyloid protein precursor. Proc Natl Acad Sci USA.

[B10] Blasko I, Marx F, Steiner E, Hartmann T, Grubeck-Loebenstein B (1999). TNFalpha plus IFNgamma induce the production of Alzheimer beta-amyloid peptides and decrease the secretion of APPs. FASEB J.

[B11] Sastre M, Dewatcher I, Landreth GE, Willson TM, Klockgether T, van Leuven F, Heneka MT (2003). Nonsteroidal anti-inflammatory drugs and peroxisome proliferator-activated receptor-gamma agonists modulate immunostimulated processing of amyloid precursor protein through regulation of beta-secretase. J Neurosci.

[B12] Vassar R (2001). The beta-secretase, BACE: a prime drug target for Alzheimer's disease. J Mol Neurosci.

[B13] Walter J, Kaether C, Steiner H, Haass C (2001). The cell biology of Alzheimer's disease: uncovering the secrets of secretases. Curr Opin Neurobiol.

[B14] McGeer PL, Rogers J (1992). Anti-inflammatory agents as a therapeutic approach to Alzheimer's disease. Neurology.

[B15] Eikelenboom P, Zhan SS, Van Gool WA, Allsop D (1994). Inflammatory mechanisms in Alzheimer's disease. Trends Pharmacol Sci.

[B16] Perry VH (2004). The influence of systemic inflammation on inflammation in the brain: implications for chronic neurodegenerative disease. Brain Behav Immun.

[B17] Sparkman NL, Martin LA, Calvert WS, Boehm GW (2005). Effects of intraperitoneal lipopolysaccharide on Morris maze performance in year-old and 2-month-old female C57BL/6J mice. Behav Brain Res.

[B18] Shaw KN, Commins S, O'Mara SM (2001). Lipopolysaccharide causes deficits in spatial learning in the water maze but notin BDNF expression in the rat dentate gyrus. Behav Brain Res.

[B19] Vernet-der Garabedian B, Lemaigre-Dubreuil Y, Delhaye-Bouchaud N, Mariani J (1998). Abnormal IL-1beta cytokine expression in the cerebellum of the ataxic mutant mice staggerer and lurcher. Brain Res Mol Brain Res.

[B20] Morris R (1984). Developments of water-maze procedure for studying spatial learning in the rat. J Neurosci Methods.

[B21] Park KS, Lee RD, Kang SK, Han SY, Park KL, Yang KH, Song YS, Park HJ, Lee YM, Yun YP, Oh KW, Kim DJ, Yun YW, Hwang SJ, Lee SE, Hong JT (2004). Neuronal differentiation of embryonic midbrain cells by upregulation ofperoxisome proliferator-activated receptor-gamma via the JNK-dependent pathway. Exp Cell Res.

[B22] Lee SM, Nguyen TH, Park MH, Kim KS, Cho KJ, Moon DC, Kim HY, Yoon DY, Hong JT (2004). EPO receptor-mediated ERK kinase and NF-kappaB activation in erythropoietin-promoted differentiation of astrocytes. Biochem Biophys Res Commun.

[B23] Culpan D, Cram D, Chalmers K, Cornish A, Palmer L, Palmer J, Hughes A, Passmore P, Craigs D, Wilcock GK, Kehoe PG, Love S TNFR-associated factor-2 (TRAF-2) in Alzheimer's disease. Neurobiol Aging.

[B24] He P, Zhong Z, Lindholm K, Berning L, Lee W, Lemere C, Staufenbiel M, Li R, Shen Y (2007). Deletion of tumor necrosis factor death receptor inhibits amyloid beta generation and prevents learning and memory deficits in Alzheimer's mice. J Cell Biol.

[B25] Gasparini L, Rusconi L, Xu H, del Soldato P, Ongini E (2004). Modulation of beta-amyloid metabolism by non-steroidal anti-inflammatory drugs in neuronal cell cultures. J Neurochem.

[B26] Yan Q, Zhang J, Liu H, Babu-Khan S, Vassar R, Biere AL, Citron M, Landreth G (2003). Anti-inflammatory drug therapy alters beta-amyloid processing and deposition in an animal model of Alzheimer's disease. J Neurosci.

[B27] Blasko I, Apochal A, Boeck G, Hartmann T, Grubeck-Loebenstein B, Ransmayr G (2001). Ibuprofen decreases cytokine-induced amyloid beta production in neuronal cells. Neurobiol Dis.

[B28] Lim GP, Yang F, Chu T, Chen P, Beech W, Teter B, Tran T, Ubeda O, Ashe KH, Frautschy SA, Cole GM (2000). Ibuprofen suppresses plaque pathology and inflammation in a mouse model for Alzheimer's disease. J Neurosci.

[B29] Sung S, Yang H, Uryu K, Lee EB, Zhao L, Shineman D, Trojanowski JQ, Lee VM, Pratico D (2004). Modulation of nuclear factor-kappa B activity by indomethacin influences A beta levels but not A beta precursor protein metabolism in a model of Alzheimer's disease. Am J Pathol.

[B30] Hwang DY, Cho JS, Lee SH, Chae KR, Lim HJ, Min SH, Seo SJ, Song YS, Song CW, Paik SG, Sheen YY, Kim YK (2004). Aberrant expressions of pathogenic phenotype in Alzheimer's diseased transgenic mice carrying NSE-controlled APPsw. Exp Neurol.

[B31] Kuperstein F, Brand A, Yavin E (2004). Amyloid Abeta1-40 preconditions non-apoptotic signals in vivo and protects fetal rat brain from intrauterine ischemic stress. J Neurochem.

[B32] Zou K, Kim D, Kakio A, Byun K, Gong JS, Kim J, Kim M, Sawamura N, Nishimoto S, Matsuzaki K, Lee B, Yanagisawa K, Michikawa M (2003). Amyloid beta-protein (Abeta)1-40 protects neurons from damage induced by Abeta1-42 in culture and in rat brain. J Neurochem.

[B33] Zou K, Gong JS, Yanagisawa K, Michikawa M (2002). A novel function of monomeric amyloid beta-protein serving as an antioxidant molecule against metal-induced oxidative damage. J Neurosci.

[B34] Hauss-Wegrzyniak B, Wenk GL (2002). Beta-amyloid deposition in the brains of rats chronically infused with thiorphan or lipopolysaccharide: the role of ascorbic acid in the vehicle. Neurosci Lett.

[B35] Liao YF, Wang BJ, Cheng HT, Kuo LH, Wolfe MS (2004). Tumor necrosis factor-alpha, interleukin-1beta, and interferon-gamma stimulate gamma-secretase-mediated cleavage of amyloid precursor protein through a JNK-dependent MAPK pathway. J Biol Chem.

[B36] Sheng JG, Bora SH, Xu G, Borchelt DR, Price DL, Koliatsos VE (2003). Lipopolysaccharide-induced-neuroinflammation increases intracellular accumulationof amyloid precursor protein and amyloid beta peptide in APPswe transgenic mice. Neurobiol Dis.

[B37] Rogers JT, Leiter LM, McPhee J, Cahill CM, Zhan SS, Potter H, Nilsson LN (1999). Translation of the Alzheimer amyloid precursor protein mRNA is up-regulated by interleukin-1 through 5'-untranslated region sequences. J Biol Chem.

[B38] Bandyopadhyay S, Hartley DM, Cahill CM, Lahiri DK, Chattopadhyay N, Rogers JT (2006). Interleukin-1alpha stimulates non-amyloidogenic pathway by alpha-secretase(ADAM-10 and ADAM-17) cleavage of APP in human astrocytic cells involving p38 MAP kinase. J Neurosci Res.

[B39] Ma G, Chen S, Wang X, Ba M, Yang H, Lu G (2005). Short-term interleukin-1(beta) increases the release of secreted APP(alpha) via MEK1/2-dependent and JNK-dependent alpha-secretase cleavage in neuroglioma U251 cells. J Neurosci Res.

[B40] Salbaum JM, Weidemann A, Lemaire HG, Masters CL, Beyreuther K (1988). The promoter of Alzheimer's disease amyloid A4 precursor gene. EMBO J.

[B41] Quitschke WW, Goldgaber D (1992). The amyloid beta-protein precursor promoter. A region essential for transcriptional activity contains a nuclear factor binding domain. J Biol Chem.

[B42] Williams T, Tjian R (1991). Analysis of the DNA-binding and activation properties of the human transcription factor AP-2. Genes Dev.

[B43] Trejo J, Massamiri T, Deng T, Dewji NN, Bayney RM, Brown JH (1994). A direct role for protein kinase C and the transcription factor Jun/AP-1 in the regulation of the Alzheimer's beta-amyloid precursor protein gene. J Biol Chem.

[B44] Hashimoto Y, Chiba T, Yamada M, Nawa M, Kanekura K, Suzuki H, Terashita K, Aiso S, Nishimoto I, Matsuoka M (2005). Transforming growth factor beta2 is a neuronal death-inducing ligand for amyloid-beta precursor protein. Mol Cell Biol.

[B45] Hensley K, Floyd RA, Zheng NY, Nael R, Robinson KA, Nguyen X, Pye QN, Stewart CA, Geddes J, Markesbery WR, Patel E, Johnson GV, Bing G (1999). p38 kinase is activated in the Alzheimer's disease brain. J Neurochem.

[B46] Giovannini MG, Scali C, Prosperi C, Bellucci A, Vannucchi MG, Rosi S, Pepeu G, Casamenti F (2002). Beta-amyloid-induced inflammation and cholinergic hypofunction in the rat brain in vivo: involvement of the p38MAPK pathway. Neurobiol Dis.

[B47] Noble F, Rubira E, Boulanouar M, Palmier B, Plotkine M, Warnet JM, Marchand-Leroux C, Massicot F (2007). Acute systemic inflammation induces central mitochondrial damage and mnesic deficit in adult Swiss mice. Neurosci Lett.

[B48] Milatovic D, Zaja-Milatovic S, Montine KS, Horner PJ, Montine TJ (2003). Pharmacologic suppression of neuronal oxidative damage and dendritic degeneration following direct activation of glial innate immunity in mouse cerebrum. J Neurochem.

[B49] Szczepanik AM, Ringheim GE (2003). IL-10 and glucocorticoids inhibit Abeta(1-42)- and lipopolysaccharide-induced pro-inflammatory cytokine and chemokine induction in the central nervous system. J Alzheimers Dis.

[B50] Jang JH, Surh YJ (2005). Beta-amyloid-induced apoptosis is associated with cyclooxygenase-2 up-regulation via the mitogen-activated protein kinase-NF-kappaB signaling pathway. Free Radic Biol Med.

[B51] Song YS, Park HJ, Kim SY, Lee SH, Yoo HS, Lee HS, Lee MK, Oh KW, Kang SK, Lee SE, Hong JT (2004). Protective role of Bcl-2 on beta-amyloid-induced cell death of differentiated PC12 cells: reduction of NF-kappaB and p38 MAP kinase activation. Neurosci Res.

[B52] Bate C, Kempster S, Last V, Williams A (2006). Interferon-gamma increases neuronal death in response to amyloid-beta1-42. J Neuroinflammation.

[B53] Hashimoto Y, Nawa M, Chiba T, Aiso S, Nishimoto I, Matsuoka M (2006). Transforming growth factor beta2 autocrinally mediates neuronal cell death induced by amyloid-beta. J Neurosci Res.

[B54] Culbert AA, Skaper SD, Howlett DR, Evans NA, Facci L, Soden PE, Seymour ZM, Guillot F, Gaestel M, Richardson JC (2006). MAPK-activated protein kinase 2 deficiency in microglia inhibits pro-inflammatory mediator release and resultant neurotoxicity. Relevance to neuroinflammation in a transgenic mouse model of Alzheimer disease. J Biol Chem.

